# How to engage patients in research and quality improvement in community-based primary care settings: protocol for a participatory action research pilot study

**DOI:** 10.1186/s40900-018-0113-3

**Published:** 2018-10-01

**Authors:** Julie Haesebaert, Isabelle Samson, Hélène Lee-Gosselin, Sabrina Guay-Bélanger, Jean-François Proteau, Guy Drouin, Chantal Guimont, Luc Vigneault, Annie Poirier, Priscille-Nice Sanon, Geneviève Roch, Marie-Ève Poitras, Annie LeBlanc, France Légaré

**Affiliations:** 1 Centre de recherche sur les soins et services de première ligne de l’Université Laval, CIUSSS de la Capitale-Nationale, Pavillon Landry-Poulin, 2525, chemin de la Canardière, Quebec City, Quebec G1J 0A4 Canada; 2 Tier 1 Canada Research Chair in Shared Decision Making and Knowledge Translation, Université Laval, Quebec, QC Canada; 3 Department of Family Medicine and Emergency Medicine, Faculty of Medicine, Université Laval, Quebec City, Quebec Canada; 4 Department of Management, Faculty of Business Administration, Université Laval, Quebec City, Quebec Canada; 5 Quebec City, Canada; 6 Centre Hospitalier Universitaire de Québec – Université Laval Research Centre, Hôpital Saint-François d’Assise, Quebec City, Quebec Canada; 7 Faculty of Nursing, Université Laval, Quebec City, Quebec Canada; 8 Department of Health Sciences, Université du Québec à Chicoutimi, Chicoutimi, Quebec Canada

**Keywords:** Primary care, Quality improvement, Patient involvement, Patient-centeredness, Patient advisory council, Participatory action research

## Abstract

**Plain English summary:**

Making primary care clinics more patient-centered is key to improving patients’ experience of care. If patients themselves were engaged in helping define priorities and suggesting quality improvements in the clinic, care would respond better to their needs. However, patient engagement is a new phenomenon, particularly in community based primary care clinics. How to engage patients in quality improvement in these clinics, or what effect this might have, is not well known. The involvement of patients needs to be adapted to the way these clinics function. The aim of this study is to create and evaluate a new model of patient engagement for quality improvement in community based primary care clinics. Patients, primary care professionals and researchers will create advisory councils in two primary care clinics in Quebec City (Canada). In each clinic, the advisory council will include 12 patients or caregivers registered at the clinic, a clinician and a clinic manager. The advisory council will meet every 6 weeks for a total of six meetings. Two patient-experts will facilitate meetings. During meetings, members of the council will list their needs in order of importance. Then they will suggest improvements in line with these needs. We will study if our advisory council model is well adapted to community based primary care settings and meets participants’ expectations. At the end of the study we will be able to offer guidance about engaging patients with health professionals in quality improvement in primary care clinics.

**Abstract:**

**Background**

Involvement of end-users, including patients, managers and clinicians, in identifying quality improvement and research priorities might improve the relevance of projects and increase their impact. Few patient engagement initiatives have taken place in community based primary care practices (CBPCPs) and best practices for engaging patients in such settings are not well defined. The aim of this pilot study is to develop and assess the feasibility of a new collaborative model of advisory council involving clinicians, managers, patients and caregivers in CBPCP to strengthen their capacity to conduct quality improvement and patient-oriented research projects.

**Methods**

We will conduct a participatory action research project in two non-academic CBPCPs in Quebec City (Canada). In each CBPCP, the advisory council will include 12 patients or caregivers, a clinician and a clinic manager. Patients or their caregivers will be identified by clinicians and contacted by patient-experts. They will be eligible if they are registered at the practice, motivated, and available to attend meetings. The council will meet every 6 weeks for a total of six meetings. Two patient-experts will guide council members to identify quality improvement priorities and patient-oriented research questions based on their experience in the clinic. They will then be supported to plan actions to target these priorities. Analysis of meetings will be based on feasibility criteria, notes by non-participant observers in log books, audio-recording of the meetings and questionnaires to evaluate council members’ perceptions and the likelihood they would engage in such councils.

**Discussion**

The results of this study will  be a model of patient engagement and a discussion of factors to improve the model to fit the needs of primary care patients and professionals. This will lay the foundation for a sustainable structure for long-term patient engagement and contribute to the development of a patient-centered and quality-improvement culture in CBPCPs.

## Background

Health professionals, organizations and policy makers in health and social fields are placing increasing emphasis on integrating the perspective and experience of patients and their families in the health care system [1–3]. This translates into a switch in focus from a paternalistic approach to integrating patients’ preferences at all levels of the healthcare process: at the point-of-care, at the organizational level and in research [4, 5]. Quality improvement (QI) is defined as the combined and unceasing efforts of healthcare professionals, patients and families, researchers, payers, planners and educators to make the changes that will lead to better patient outcomes, better system performance and better professional development [6]. QI activities targeting topics relevant to patients could improve quality and safety of care in ways that mean more to patients and improve their care experience. When patients and the public are involved in setting QI priorities, they raise different topics to those raised by health professionals without patients’ perspectives [7]. However, few comparative data are available for rigorous measurement of the impact of engaging patients in QI [8, 9]. The literature also underscores discrepancies between patient priorities and published research [10, 11] and advocates refocusing research funding on topics that are more meaningful to patients, clinicians and policy makers (hereafter “knowledge users”) [12, 13]. Engaging knowledge users early on to sound out their expectations and priorities may improve project relevance and lead to results having a greater impact on clinical practices and patient health outcomes [14, 15].

Patient and end-user involvement is widely promoted in community based primary care practices (CBPCP) [16–18]. In Canada, the College of Family Physicians of Canada promotes patient centeredness and “continuous quality improvement and patient feedback” in its Patient’s Medical Home model [18], as does the US in the Patient-Centered Medical Homes model supported by the Affordable Care Act [19]. However, practices across Canada and US do not fully align with these models yet [20, 21]. Inviting groups of patients to meet on a regular basis to provide feedback on their experience of care in the CBPCP could represent a suitable model of long term partnership. Reported experiences of sustainable patient involvement initiatives at the organizational level mostly target academic settings and remain scarce in CBPCPs [22–24]. In the US, patient advisory councils remain optional for accreditation of Patient-Centered Medical Homes [17]. In the UK, “patient reference groups”, which aim to involve patients in decision making to improve services in primary care practices, expanded when incentives were offered [25]. However, the wide variation in their activity, functioning and organization reflects a wide variation in the level of patient involvement [26, 27]. Some have produced relevant innovations through meaningful involvement, but others have faced recruitment and sustainability issues, and in some cases the patients played a passive role resulting in little change in practices [26–28]. Evidence regarding the impact of such patient councils in primary care is limited and needs to be expanded to evaluate their potential benefits and understand the mechanisms of their effects [22, 28–30]. Reported experiences concur on the need for a rigorous framework to underpin the development of councils, with clear objectives, sufficient resources, appropriate support and incentives, training for participants and true decision making capabilities to limit the risk of tokenism [26–28, 30, 31]. A structured and rigorous evaluative approach should also be integrated into the implementation of the councils to demonstrate their impact.

We hypothesized that a model of engagement conceptualized by and for knowledge users, including patients, clinicians and clinic managers, could be feasible in CBPCPs and result in patient-oriented QI projects. This pilot study aimed to design and assess the feasibility of a new collaborative model of advisory councils involving health professionals, managers, patients and caregivers in CBPCPs to strengthen their capacity to conduct QI and patient-oriented research projects. The secondary aim is to explore the impact of sex and gender on participation in these councils and QI priorities.

## Methods

### Design

**Fig. 1:**
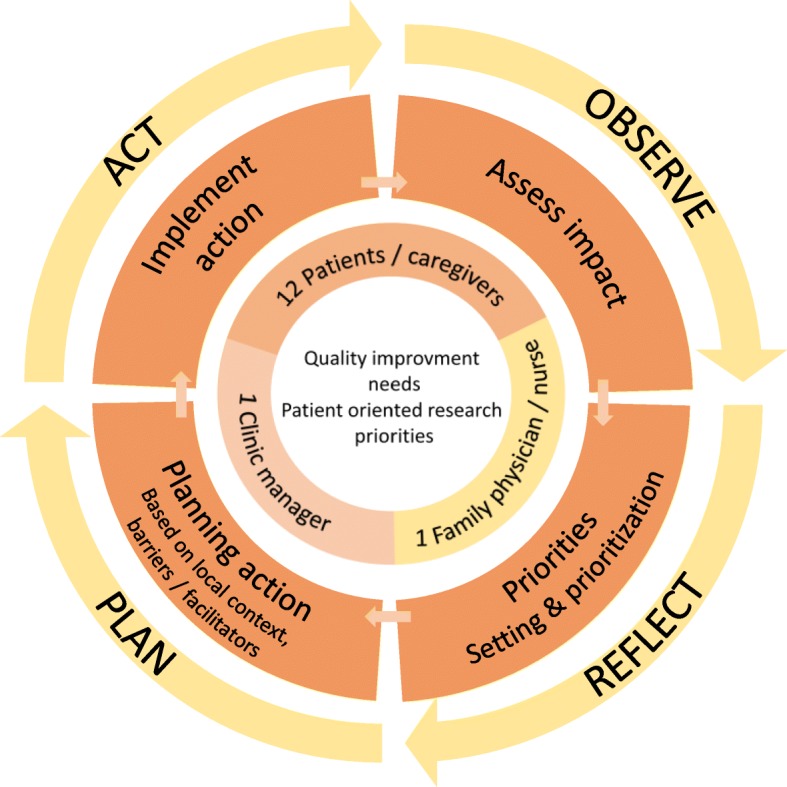
The EQUIPPS (*Équipes Patients, Proches aidants, Soignants*, or in English, “patient, caregiver and healthcare provider teams”) cycle from the inside out: needs and priorities, council members, project development cycle, Participatory Action Research cycle (framework)

We will develop the model with knowledge users using a participatory action research (PAR) approach. PAR is a systematic inquiry that implies a mutually respectful partnership between researchers and participants. Participants are considered as equals by researchers rather than as subjects of research. Success in this approach depends on the quality of the collaboration, commitment of participants, mutual education incorporating a democratic sharing of expertise and knowledge, and targeting a topic that is relevant to all participants [32, 33]. PAR is described as an iterative process following cycles of the following repeating steps: Reflect, Plan, Act and Observe (Fig. 1) [32]. We will ensure that the development of our model is participatory and collaborative at each step. The project steering committee is an interdisciplinary team of researchers from health and social sciences, including family physicians, nurses, a coordinator from the Quebec’s practice based research network (QPBRN) providing support for research to CBPCPs in Quebec, and a patient with research experience (JFP), all of whom were involved in the study design and will be involved in decision making as the study progresses.

### Patient-expert participation

This study will be conducted in partnership with four patients, all trained in the science of patient partnership in research [34–36]. The training they received consisted of three modules on 1) the basis, 2) the first steps and 3) consolidation of patient partnership in research. Moreover, they have all had at least one year of experience in research participation. We refer to them hereafter as “patient-experts”. They were recruited either through having being partners in other projects with our team or were referred by the Quebec SPOR SUPPORT Unit (http://unitesoutiensrapqc.ca). One patient-expert (JFP) is co-principal investigator of the project and member of the steering committee, and contributed to  writing this study protocol by revising the protocol draft. Three other patient-experts were also involved as soon as the funding was obtained. To achieve the study objective regarding the impact of sex and gender on involvement, patient-experts will function in mixed-gender tandem in all their activities. All four patient-experts will be involved in recruiting council members, designing content and tools and facilitating meetings. Their perspective and interactions with the study coordinator will also contribute to shaping the analysis plan. They will contribute to discussion and interpretation of the study results and will be involved as co-authors in all publications of study results by critically revising the article. During the study, they will be supported by the study coordinator (JH) who will set up the agenda and activities of the councils meetings with them and ensure all practical aspects of organization. Patient-experts and the study coordinator will meet before each council meeting and as needed throughout the study period.

### Study setting

In the province of Quebec (Canada), most CBPCPs are accredited as family medicine groups (GMFs or *Groupes de Médecine de Famille*) [37], consisting mainly of family physicians, primary care nurses and nurse practitioners. The study will take place in two privately owned accredited GMFs in Quebec City (QC, Canada). The first CBPCP (“CBPCP-A”) is part of a group of three CBPCPs located in Quebec City and Montreal. IS, co-investigator of this study, practices as a family physician in this CBPCP-A. The second CBPCP (“CBPCP-B”) opened in May 2017, three months before the study received ethics approval. CBPCP-B is an accredited “Super-clinic”, which means it provides care 14 hours/day, seven days a week including for non-enrolled patients (walk-in) and with higher technical levels of care [38]. Characteristics of the two participating CBPCPs are presented in Table 1. Both clinic directors had given their written commitment to participate in the study before the funding was obtained. This project was the first and only on-going research and QI project held in the participating CBPCPs. No other patient involvement activity had taken place in these CBPCPs either.

**Table 1:** Participating Community-Based Primary Care Practices

Status	CBPCP-A	CBPCP-B
	Family Medicine Group	Superclinic*
Number of family physicians practicing at the clinic	11	28
Other professionals practicing at the clinic	3 clinician nurses	3 clinician nurses
	1 social worker	1 social worker
	1 clinic coordinator	
Number of enrolled patients	12,000	19,770
Services provided in the CBPCP	public family practice care	public family practice care
	private family practice care	private family practice care
	physiotherapy	urgent care
	orthopedic care	perinatal care
	otolaryngology care	musculoskeletal medicine

*CBPCP* Community-Based Primary Care Practices

^*^A superclinic (“GMF reseau”) is a type of CBPCP specific to Quebec. It provides walk-in care and offers more admission hours than other CBPCPs

### Participants

**Fig. 2:**
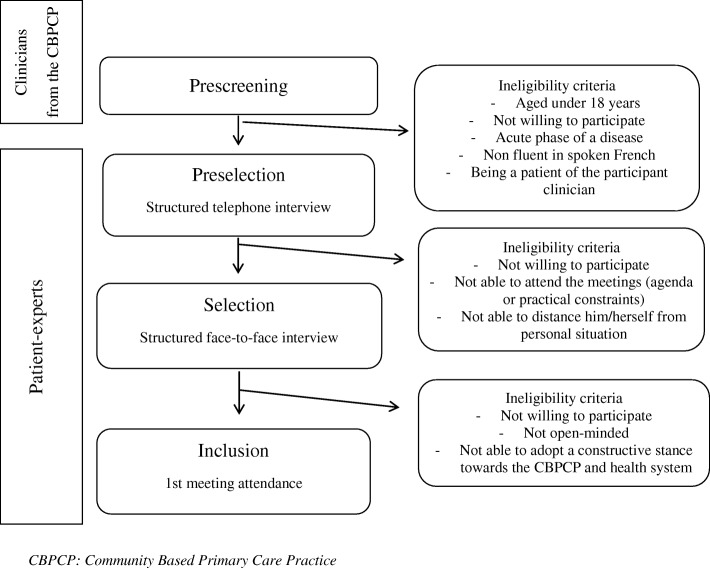
Flow chart of recruitment of patients and caregivers

Based on recommendations in the available literature on conducting patient advisory councils, each council will consist of 12 patients and/or caregivers, one health professional and one CBPCP manager [23, 24, 26, 39, 40]. Inclusion criteria for all council members are broad and mainly based on willingness to participate in order to recruit a large variety of council members, to improve feasibility, and to fit in with the day-to-day realities of non-academic CBPCPs. Specifically, managers can be from either medical or administrative backgrounds and must be responsible for managing the participating CBPCP during the study period. Health professionals must be practicing at the participating CBPCP as their main activity, either as a family physician or a primary care nurse. The CBPCP manager and the health professional will be recruited during the kick-off meeting in each CBPCP based on their interest in the project and availability to attend all meetings. Patients have to be registered at the CBPCP, to be 18 years old and over, to have the capacity to stand back from their own condition and illness, to demonstrate interest in improving common wellness, and be willing and available to attend the meetings. Patients cannot participate if they are under care of the health professional attending the meetings, if they are in an acute phase of their disease, if they have had a conflict with the clinic or behaved inappropriately with clinic staff and if they are not fluent in French. No restrictions apply regarding clinical condition and illness. Caregivers are defined as relatives of a patient registered at the participating CBPCP. Inspired by an interview grid designed for patient-partner recruitment [41], the recruitment process will follow three steps: 1) patients and caregivers will be identified according to inclusion and exclusion criteria by health professionals of the CBPCP who are not directly involved in the advisory council, 2) patients and caregivers will be interviewed by a patient-expert over the phone in order to explain the purpose and processes of the study, check inclusion criteria, patient motivation and availability to participate, 3) eligible patients and caregivers will be interviewed face-to-face by two patient-experts to ensure that they understand the study aims as well as the responsibilities and commitments the study will entail. At least one caregiver per CBPCP will be selected and a balanced representation of male and female council members sought. The selection process is presented in Fig. 2.

### The EQUIPPS model

We referred to the councils as « Équipes Patients, Proches aidants, Soignants » (in English, “patient, caregiver and healthcare provider teams”). The first version of the EQUIPPS model was co-designed by researchers (FL, GR, AL, HLG, MEP, SGB, JH), patient-experts (JFP, AP, PNS, LV) and a family physician (IS). During the study, the council model will be refined and tailored to fit the needs of its members.

### Theoretical framework

Guided by the patient engagement framework [5, 42, 43], the EQUIPPS councils designed in this study aimed to create partnership at the organizational level, involving patients in priority setting and decision-making about QI and patient-oriented research projects [5]. Practical aspects of the EQUIPPS councils were defined based on a review of experiences reported in the literature [23, 24, 26, 39, 40] and on the Institute for Patient and Family-Centered Care guidelines [44, 45]. Our approach was also guided by the patient engagement framework and the capacity development framework proposed by the Canadian Strategy for Patient Oriented Research (SPOR) [1, 46].

### Functioning

The EQUIPPS councils will meet for six 90-minute meetings which will take place every six weeks, depending on members’ availability, over a 12-month period. A meeting will occur if the manager, the health professional and a minimum of six patients or caregivers are available to attend. Meetings will be held in each CBPCP’s meeting room in the evening. Every effort will be made to promote a friendly and collaborative atmosphere during the meetings and snacks will be provided. In each CBPCP, a mixed tandem of patient-experts (PNS and LV in CBPCP-A and AP and JFP in CBPCP-B) will facilitate the meetings, promoting active collaboration and exchange among councils members. Facilitators will guide discussions to ensure that all council members freely express their points of view, to avoid any leadership or power takeover by one member, or other disruption that would bias the interchange.

### Content

Table 2 presents the meeting plans and Fig. 1 shows the steps that council members will follow in the study process. The first meeting will include a one-hour training by patient-experts in each clinic on the organization of the healthcare system, patient engagement in care, QI and research, so that all council members have the same common core knowledge before engaging in the process. Training materials were developed by the patient-experts supported by a researcher (JH), and are based on Quebec Ministry of Health documents [37] and on literature on patient engagement and QI [1, 5, 42, 46]. The training content has been reviewed by the steering sommittee. In the next meetings, council members will reflect on and discuss QI priorities and patient-oriented research projects that would be relevant at the clinic level, according to their experience of care and their needs. They will be supported in this approach by activities to help highlight and prioritize topics, such as a “brainwriting” activities and prioritization activities guided by the Child Health and Nutrition Research Initiative criteria [47] to select the theme that is the most relevant to them. According to their progress, they will define a structured QI or patient-oriented research question in line with the selected theme, discuss potential actions to address this question and identify barriers and facilitators to be taken into account. In accordance with PAR principles, the facilitators will guide and support council members to target these objectives, but the council will proceed at its own pace. Activities or resources will be adapted to the needs and demands of the council members from one meeting to the next. For instance, if necessary, stakeholders or researchers could be invited to the meetings to present scientific evidence on a specific topic. If a topic leads to a research project, the council could be put in touch with a research team from the QPBRN to further its goals. The last meeting will consist of a focus group to assess council members’ experience of being on the council.

**Table 2:** Planned content of meetings co-designed by patient-experts, family physicians and researchers

Meeting	Time frame	Content	Description of activities	Research project – data collection
1	Week 1	Introductory meeting	Short training on health system, QI process and POR	Informed consent questionnaires
			Definition of rules and functioning of council	Audio-recording
				Non-participant observation
2	Week 6	Topic identification	Brainstorming activities (half group and whole group)	Audio-recording
				Non participant observation
3	Week 12	Prioritization of topics	Analysis according to impact and feasibility	Audio-recording
				Non-participant observation
			Choice of a topic	
4	Week 18	Action planning	Definition of the question to be addressed based on the chosen topic	Audio-recording
				Non-participant observation
			Analysis of local context, barriers and facilitators to implementing actions	
5	Week 24	Action planning	Definition of actions to target identified priorities	Audio-recording
				Non-participant observation
6	Week 30	Wrap-up meeting	Perception and experience of participants on the council	Questionnaires
				Audio-recording Non-participant
			Strengths and limitations of the council	
				Observation

*POR* Patient-oriented research, *QI* Quality improvement

### Outcomes criteria

The feasibility of implementing our council model in the participating CBPCPs will be assessed based on the following criteria [48]:

Process issues: retention of CBPCPs and council members throughout the study; number of meetings planned and held during the 12-month study period and attendance of council members;

Resource issues: time required to recruit council members and organize meetings; communication between research team and council members; and resources needed to organize and hold all meetings;

Management issues: challenges between study personnel, clinics and council members, challenges in interactions between the council members during the meetings; capacity to overcome those challenges;

Scientific issues: QI and patient-oriented research topics that are identified during the meetings, projects and actions that are shaped around these topics.

We will also assess the perceptions of council members on their involvement, the barriers and facilitators for their involvement in the councils, interactions and leadership relationships between council members.

### Data collection

Multiple data sources will be used to collect the data. The study coordinator (JH) will collect feasibility data in a study log book, including recruitment time, number of meetings, management issues with CBPCPs and council members. During the meetings, data will be collected both through ad-hoc questionnaires and qualitative data collection process. Council members will fill out a sociodemographic questionnaire at the beginning of the study, and a questionnaire on their perception and motivations to involve in the council at the beginning of the first meeting and at the end of the last meeting. Content of all questionnaires is detailed in Table 3. These questionnaires have been developed for the present study and were guided by existing surveys on perceptions of patient involvement and patient advisory councils [44, 45, 49]. Content and form have been validated by the steering committee and patient-experts. The first questionnaire has 16 questions, including open-ended questions, multiple choice questions and statement questions with ordinal 0–10 scales or Likert scales. The first eight questions are on patient engagement, including willingness to engage with researchers, motivation for engaging in this research, and knowledge about patient-oriented research. The second eight questions are on participation in the council, including understanding of the objectives, perceptions of their role, motivation to participate and priority topics. The final questionnaire contains the same questions with the addition of questions on their perceptions of the impact of the council and barriers and facilitators to sustaining their involvement over a long term period. All discussions at the meetings will be audio-recorded and transcribed verbatim. The study coordinator (JH) and QPBRN coordinator (SGB) will attend each meeting as non-participant observers. At each meeting, the non-participant observers will fill out a log book to collect qualitative data with a structured grid on meeting processes, verbal and non-verbal behaviors, ideas developed during the meetings and interactions between council members and facilitators. Observations will include a sex and gender perspective for each topic in the log book. At the end of each meeting, the two facilitators will complete the log books with their additional observations. For the last meeting (a focus group), a semi-structured interview grid will be used to frame discussion on members’ experience of being on the council. Facilitators will guide discussions toward several predefined topics: members’ motivation and expectations of such a council, their perceptions of patient involvement and patient-oriented research, their experience, the barriers and facilitators to their involvement in the councils and their perception of the impact of the council on the CBPCP during the study period.

**Table 3:** Content of the questionnaires used in the study

	Patients	Clinic manager and health professional
Socio-demographic questionnaire	- Gender	- Gender
	- Age	- Age
	- Education	- Occupation/position in the CBPCP
	- Occupation	
		- Family situation
		- Year started at the CBPCP
	- Family situation	- History of participation in research, QI or patient engagement activities in the CBPCP or in other institutions
	- Zip code of living area	
	- Year of registration in the CBPCP	
	- Chronic diseases	
	- History of participation in other user committees or associations (in the health field or in other fields)	
Inclusion questionnaire on perceptions (before the first meeting)	- POR knowledge, definition, history of participation in POR projects, barriers and drivers to POR participation, beliefs about impact of POR	- POR knowledge, definition, history of participation in POR project, barriers and drivers to POR participation, beliefs about impact of POR
	- Belief in ability to identify QI and research priorities, identified QI or research priorities	- Belief in ability to identify QI and research priorities, identified QI or research priorities
		- Knowledge and understanding of councils’ objectives, of the role they will play in the council, drivers to their participation
	- Knowledge and understanding of councils’ objectives, of the role they will play in the council, drivers to their participation	
		- Beliefs about gender impact on participation in the councils
	- Beliefs about sex/gender impact on participation in the councils	
End-of-study questionnaire on perceptions	- POR knowledge, definition, history of participation in POR project participation, barriers and drivers for POR participation, beliefs about impact of POR	- POR knowledge, definition, history of participation in POR project participation, barriers and drivers for POR participation, beliefs about impact of POR
	- Belief in ability to identify QI and research priorities, identified QI or research priorities	- Belief in ability to identify QI and research priorities, identified QI or research priorities
	- Satisfaction about their participation in the council, perceived impact of council on CBPCP, perceived role played in the council, strengths and weaknesses of the council	- Satisfaction about their participation in the council, perceived impact of council on the CBPCP, perceived role played in the council, strengths and weaknesses of the council
	- Perceived impact of sex/gender on the council	- Perceived impact of sex/gender on the council

*CBPCP* Community Based Primary Care Practice, *POR* Patient oriented research, *QI* Quality Improvement

Full questionnaires are available in French on request to the corresponding author

### Analysis plan

All analyses will be performed at the clinic level and combined. Results from both CBPCPs will be discussed and contrasted to identify common topics and specificities of the care setting and context.

We consider a priori that implementation of our council model can be considered feasible if we reach the following thresholds, defined before study begins by the steering committee and the patient experts: 1) Process issues: the two enrolled CBPCPs are retained for the full duration of the pilot study; over the 12-month study period at least four out of the six planned meetings are held, and at least six patients, the clinic manager and the health professional attend each meeting. 2) Resource issues: total time to complete recruitment of council members is under  six months; the CBPCPs are able to respond to the study coordinator’s requests to organize meetings; resources needed to organize meetings and compensate council members are covered by the study funding. 3) Management issues: the research team receives no complaints from clinics, patient-experts or council members about the functioning and agenda of the meetings; patient-experts do not encounter difficulties in facilitating the meetings. These outcomes will be collected by study coordinator in a log book based on interactions with council members and patient- experts during and between the meetings (face-to-face, phone or email interactions). 4) Scientific issues: each council comes to a consensus on at least one QI topic or patient-oriented research question to be addressed.

We will describe sociodemographic characteristics of council members, their responses to questionnaires on their perceptions using frequencies and proportions for categorical data, or means and standard deviations or median and interquartile range (according to distribution) for continuous data. We will compare data from the initial and final questionnaires on perceptions of council members with comparative tests for paired data; Cochran Q test will be used to compare categorical data and non-parametric Wilcoxon signed rank test will be used to compare ordinal quantitative data. All *p*-values will be two-tailed and considered as statistically significant when ≤0.05. Analysis will be performed by JH using the SAS 9.4 software.

We will triangulate methods, data collection tools, data sources and observers. Notes from the log books of both observers will be combined. Any discrepancy between the two will be discussed, and if need be, resolved with the patient-experts who facilitated the meetings. We will conduct an inductive thematic analysis of qualitative data collected in the log books, verbatim transcripts of audio recordings of meetings, materials used during the meetings and responses to open-ended questions to analyse QI and patient-oriented research ideas, council members’ perceptions, barriers and facilitators to engaging in the councils and interactions during the meetings. Analysis will be conducted throughout the study by JH using the N-Vivo software. Inferences from the qualitative analysis will be combined with results of the quantitative analysis of questionnaires.

A sex-and-gender analysis will be carried out by describing sex-disaggregated data for all pre-specified outcomes, and exploring whether sex or gender influenced all dimensions of the study. The gender analysis will be guided by the framework proposed by Morgan et al. [50] describing four domains: resources, division of labor and responsibilities, social norms, and decision-making. These four domains will be developed to analyse how gender affected the content of the study (council functioning: involvement in the process, interactions between individuals, speaking time, priorities suggested by individuals and perceptions), the research process (study coordination, data collection and analysis, council facilitation) and outcomes achieved (QI or research priorities, actions taken by the councils).

All results will then be discussed by all members of the research team, including members of the steering committee and the four patient-experts, to reflect on and discuss barriers and facilitators to implementing such councils on a long-term basis.

## Discussion

By the end of the study, we hope to be able to propose a model of collaborative engagement of patients/caregivers, health professionals and clinic managers in CBPCP settings on a regular and sustainable basis. We will discuss the feasibility of this model, and based on barriers and facilitators identified, we will propose ways to improve it. We will also describe QI and research priorities identified at the clinic level and describe actions planned or implemented to address these topics.

Our study has some limitations. Firstly, the participation of the CBPCPs in this pilot feasibility study is voluntary and the two CBPCPs have characteristics that limit the generalizability of the results. They are high-volume clinics in urban areas, thus not representative of all CBPCPs in the province of Quebec. Also, one of the co-researchers of the study practices in CBPCP-A. Secondly, the selection of the patient members of the councils by clinicians of the CBPCPs might biase it toward inclusion of more highly-educated patients or those with a more favorable attitude toward the clinic. However, the aim of our pilot study was not to be representative of the whole landscape of primary care clinics and patients but to co-design a model with patients and primary care clinic professionals. Assessment on a larger and more diversified sample will be conducted in the next development stage. Moreover, since implementing patient councils is not mandatory in Canadian CBPCPs, the willingness of clinics and participants is a prerequisite for setting them up. Our study addresses the main limitations of other patient council initiatives reported in the literature. Our model is designed according to a theory-based framework for patient engagement [5] and training will be provided to all council members during the first meeting to strengthen their capacity to discuss priorities. The active participation of four trained and experienced patient-experts increases the likelihood that we will succeed in establishing a meaningful partnership with council members. The PAR approach will enable us to improve the model to fit the needs of end-users and improve its relevance. The co-design of the model also ensures its local applicability and sufficient adaptability to be transferable to other CBPCPs.

The results will contribute to defining the best model to engage patients in the long term in CBPCPs and to improving understanding of the mechanisms of patient engagement. One of the ultimate purposes of our project is also to foster a QI and patient-centeredness culture in CBPCPs. Creating and sustaining patient partnerships in primary care practices is a necessary condition to achieving the healthcare quadruple aim, namely, improved patient experience of care, improved health outcomes, improved clinician experience and reduced costs [51]. This pilot study is the initial exploratory stage in the development and assessment of the impact of our councils. Following Medical Research Council guidance [52], in the next phases we will rigorously evaluate the impact of the implementation of these councils in CBPCPs on the experience of care in a larger scale comparative study.

## Abbreviations

CBPCP: Community based primary care practice
GMF: Family medicine group (Groupe de Médecine de Famille)
QI: Quality improvement
